# Associations between serum micronutrients and all-cause, cancer, and cardiovascular mortality in a national representative population: Mediated by inflammatory biomarkers

**DOI:** 10.1016/j.redox.2025.103573

**Published:** 2025-02-26

**Authors:** Chunliang Liu, Harrison Wongsonegoro, Tianchen Sheng, Hao Fan, Jianjun Zhang

**Affiliations:** a Department of Epidemiology, Indiana University Richard M. Fairbanks School of Public Health, Indianapolis, IN, USA; b Department of Gastroenterology, The Second Hospital of Shanxi Medical University, Taiyuan, People's Republic of China; c Department of Epidemiology and Biostatistics, Indiana University School of Public Health, Bloomington, IN, USA; d Department of Surgery, Indiana University School of Medicine, Indianapolis, IN, USA; e Indiana University Melvin and Bren Simon Comprehensive Cancer Center, Indianapolis, IN, USA

**Keywords:** Micronutrients, Inflammation, Cancer, Cardiovascular disease, Mortality

## Abstract

**Background:**

Micronutrient intake was inversely associated with cancer and cardiovascular risk in previous studies, but obtained results were inconsistent and the biological mechanisms for this potential protective effect remain elusive. Therefore, we investigated the associations of serum vitamin C, 25(OH)D, α-tocopherol, β-carotene, lycopene, folate, and iron with all-cause, cancer, and cardiovascular mortality. We further evaluated whether these associations were mediated through altered inflammatory responses.

**Methods:**

Data were obtained from 11,539 participants aged ≥40 years in the National Health and Nutrition Examination Survey (NHANES) in 2001–2006 and 2017–2018. Mortality status of the participants with an average follow-up of 10.5 years was ascertained from the linked mortality files of the National Death Index. Cox proportional hazards regression was performed to evaluate mortality risk in relation to serum micronutrients, while mediation analysis was used to assess the mediating effects of serum C-reactive protein and white blood cell count on the associations of interest.

**Results:**

After adjustment for confounders, serum levels of vitamin C, 25(OH)D, β-carotene, and lycopene were associated with a reduced risk of death from all causes, cancer, and cardiovascular disease. For example, HRs (95 % CIs) for quartiles 2, 3, and 4 vs. quartile 1 of 25(OH)D were, respectively, 0.72 (0.62, 0.83), 0.70 (0.62, 0.79), and 0.66 (0.56, 0.78) (p-trend: <0.0001) for all-cause mortality, 0.68 (0.52, 0.91), 0.54 (0.39, 0.73), and 0.48 (0.32, 0.71) (p-trend: 0.0001) for cancer mortality, and 0.64 (0.50, 0.83), 0.66 (0.53, 0.83), and 0.59 (0.42, 0.82) (p-trend: 0.0012) for cardiovascular mortality. Additionally, serum C-reactive protein significantly mediated 5.3%–20.4 %, 4.5%–18.1 %, and 3.3%–15.7 % of the associations of vitamin C, 25(OH)D, β-carotene, and lycopene with all-cause, cancer, and cardiovascular mortality, respectively.

**Conclusion:**

This study suggested that serum levels of several antioxidants and vitamin D were inversely associated with all-cause, cancer, and cardiovascular mortality, mediated in part by mitigated inflammatory responses.

## Highlights

• Serum vitamin C, 25(OH)D, β-carotene, and lycopene were inversely associated with all-cause, cancer, and cardiovascular mortality.
• Most above-mentioned associations exhibited a threshold effect.
• These beneficial effects were in part mediated by altered inflammatory response.
• Observed novel findings were based on data from a national representative population.

## Graphical abstract

**Figure:**
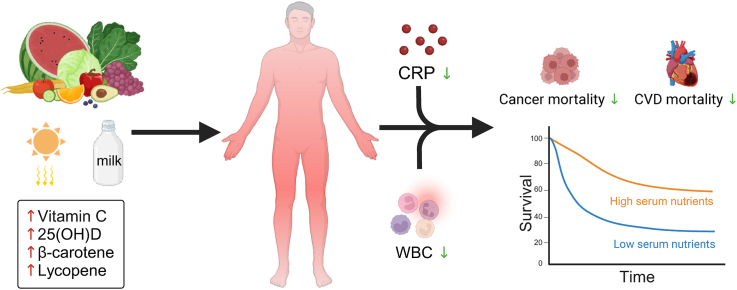

## 1 Introduction

Cancer and cardiovascular disease (CVD) are the leading causes of death among populations worldwide [1]. These two types of diseases share proinflammatory risk factors, including cigarette smoking, diet low in vegetables and fruits and high in red meat and saturated fat, and obesity [2]. Emerging experimental evidence suggests that inflammation is implicated in the pathogenesis and progression of both cancer and CVD [2,3]. Moreover, the circulating levels of inflammation biomarkers [e.g., C-reactive protein (CRP) and white blood cell (WBC) count] were positively associated with cancer and CVD mortality [[4], [5], [6], [7]]. It is thus possible to reduce the risk of cancer and CVD through implementing interventions for dietary and other risk factors that induce and promote chronic inflammation.

Micronutrients (e.g., vitamins and minerals) are critical for the modulation of oxidative stress and persistent inflammation, major drivers of carcinogenesis and cardiovascular pathogenesis [8]. Epidemiologic studies have shown that dietary intake or supplementation of vitamin C, vitamin D, α-tocopherol, and folate was associated with a reduction in CRP levels [[9], [10], [11], [12]]. Similar inverse associations were observed for serum indicators of micronutrients, including vitamin C, vitamin D, β-carotene, lycopene, and folate [13,14]. However, the roles of micronutrients in the regulation of inflammation may be complex as vitamin A supplementation increased serum levels of CRP but reduced the levels of tumor necrosis factor-alpha [15].

Numerous studies have evaluated the effects of micronutrients on all-cause, cancer, and cardiovascular mortality [16,17]. Significantly inverse associations with these death outcomes were observed for the dietary intake and serum concentrations of vitamin C, vitamin D, and folate [14,17,18], although the patterns of the associations are inconsistent across previous studies [19,20]. Both inverse and null associations with all-cause, cancer, and cardiovascular mortality were reported for vitamin E and β-carotene [[21], [22], [23]]. The discrepant findings obtained across various studies highlight the need to investigate the potential beneficial effects of micronutrients on all-cause and cause-specific mortality and to elucidate their underlying biological mechanisms in additional large, well-designed epidemiological studies.

The primary objective of the present study was to evaluate the associations of serum micronutrients [i.e., vitamin C, 25-hydroxyvitamin D (25(OH)D), α-tocopherol, β-carotene, lycopene, folate, and iron] and inflammatory biomarkers (i.e., CRP and WBC count) with all-cause, cancer, and cardiovascular mortality in a nationally representative population in the US. In addition, this study also sought to investigate whether and to what extent these inflammatory biomarkers mediate the associations of interest.

## 2 Materials and methods

### 2.1 Study population

Data were sourced from the National Health and Nutrition Examination Survey (NHANES) for the cycles 2001–2006 and 2017–2018. These cycles were selected because they collected data on both serum micronutrients and inflammatory biomarkers used in the present study. Initiated in 1999, NHANES is a nationally representative survey of the health and nutritional status of the U.S. civilian noninstitutionalized population every two years [24]. The survey design and data collection methods for NHANES have been described in detail previously [25]. The protocols of NHANES were approved by the National Center for Health Statistics Ethics Review Board and written informed consent was obtained from all participants [26].

A total of 40,763 participants were enrolled in NHANES during the 2001–2006 and 2017–2018 cycles. We excluded 29,224 individuals who were younger than 40 years old (n = 27,040), were pregnant (n = 10), did not have data on body weight (n = 843) or covariates (n = 1299), or were followed for less than one month (n = 32). After these exclusions, 11,539 participants were available for the final analysis (Supplementary Fig. 1).

### 2.2 Data collection

Data on demographic characteristics, lifestyle factors, and medical history were collected from individuals through a well-developed questionnaire. Demographic variables included age, sex, and race/ethnicity (non-Hispanic white, non-Hispanic black, Mexican American, and other races). Lifestyle factors examined were body mass index (BMI: <25 kg/m^2^, 25–30 kg/m^2^, and ≥30 kg/m^2^), educational attainment (high school graduate or below, some college or above), cigarette smoking (never, former, and current smokers), and alcohol intake (never, former, and current drinkers). Medical history considered included diagnoses of cancer, hypertension, diabetes, heart failure, coronary heart disease, angina pectoris, myocardial infarction, and stroke.

Mortality status of NHANES participants was extracted from the linked mortality files of the National Death Index. The linkage methods were described in detail elsewhere [27]. In the present study, the outcomes of interest were all-cause, cancer, and cardiovascular mortality. Cardiovascular mortality was defined as death from heart disease and cerebrovascular disease. Follow-up time was calculated from the examination date at the mobile examination center to either the date of death or December 31, 2019, whichever came earlier.

### 2.3 Laboratory measurements

#### 2.3.1 Serum micronutrients

Instructions for specimen collection and processing were described in the NHANES Laboratory Procedure Manual [28]. Methods for measuring serum levels of selected micronutrients were isocratic high-performance liquid chromatography (HPLC) with electrochemical detection at 650 mV for vitamin C, liquid chromatography-tandem mass spectrometry (LC-MS/MS) for 25(OH)D, HPLC with photodiode array detection for α-tocopherol, β-carotene, and lycopene, and the FerroZine reagent on a Roche Cobas 6000 analyzer for iron. Serum folate was quantified using the Bio-Rad Quantaphase II Folate radioassay for the cycles 2001–2006 and isotope-dilution LC-MS/MS for the cycles 2017–2018. Serum folate levels determined by the two methods have been corrected for calibration bias in the dataset [29].

#### 2.3.2 Serum inflammatory biomarkers

Serum CRP was measured with latex-enhanced nephelometry and WBC count was determined using the Beckman Coulter method [28].

### 2.4 Statistical analysis

As NHANES employed a complex, multistage probability sampling design, sample weights and design variables (SDMVPSU and SDMVSTRA) were applied in our statistical analysis [30]. Serum micronutrients and inflammatory biomarkers were log-transformed to approximate normal distributions prior to analysis. Demographic characteristics, lifestyle factors, medical history, WBC count, and serum micronutrients of participants were compared across quartiles of serum CRP levels. Continuous variables are presented as weighted means ± standard error (SE) and were compared using sampling-weighted ANOVA. Categorical variables are reported as unweighted counts with weighted proportions and were compared using sampling-weighted Rao-Scott chi-square tests.

Pearson correlations between serum levels of micronutrients and inflammatory biomarkers were calculated using the %SURVEYCORRCOV SAS Macro [31]. Linear regression was performed to evaluate the associations between serum micronutrients and inflammatory biomarkers. Logistic regression was utilized to estimate odds ratios (ORs) and 95 % confidence intervals (CIs) for serum CRP levels ≥3.0 mg/L in relation to serum micronutrients [32]. In the logistic regression analysis, each of serum micronutrients was categorized into quartiles, with participants in the lowest quartile as the reference group.

Cox proportional hazards regression was employed to estimate hazard ratios (HRs) and 95 % CIs for all-cause, cancer, and cardiovascular mortality in relation to serum micronutrients and inflammatory biomarkers. In the Cox proportional hazards regression analysis, each of serum micronutrients and inflammatory biomarkers was divided into quartiles and participants in the lowest quartile were treated as the reference group. The proportional hazards assumption was tested with Schoenfeld residuals for all models constructed, and none of them violated the assumption. Linear trend tests across quartiles of serum micronutrients and inflammatory biomarkers were conducted by using the median values of each quartile as a continuous variable in both logistic regression models and Cox proportional hazards models. Nonlinear dose-response associations of interest were analyzed using restricted cubic spline function with three knots located at the 5th, 50th, and 95th percentiles [33]. Multivariable models were adjusted for age, sex, race, BMI, education, cigarette smoking, alcohol intake, and medical history, which were selected as confounders based on the results of previous studies [14,34].

The mediation effects of inflammatory biomarkers on the associations between serum micronutrients and all-cause, cancer, and cardiovascular mortality were evaluated using the R package ‘mediation’ [35], adjusting for the confounders mentioned above. The proportion of mediation was calculated by dividing the estimate of the indirect effect by that of the total effect. An observation of significant indirect effect, significant total effect, and a positive proportion of the mediated effect indicates the presence of mediation effect [34]. Sensitivity analyses for the associations considered were performed by excluding participants with less than two years of follow-up to explore the possibility of reverse causality. All statistical analyses were performed with SAS Survey procedures and R. A two-sided p-value of <0.05 was considered statistically significant.

## 3 Results

### 3.1 Characteristics of participants

The characteristics of participants across quartiles of serum CRP levels are presented in Table 1. Individuals with higher serum levels of CRP tended to be older, female, obese, less educated, current smokers, and alcohol abstainers (all p < 0.0001). Cancer, hypertension, myocardial infarction, stroke, and diabetes were more common among persons with higher serum levels of CRP (all p < 0.0001). Serum levels of all micronutrients, except for α-tocopherol, exhibited a monotonic decline across increasing quartiles of serum CRP (all p < 0.0001).

**Table 1:** Characteristics of participants in the National Health and Nutrition Examination Survey in 2001–2006 by quartiles of serum C-reactive protein.

Characteristics	Serum C-reactive protein (mg/L)				
	Q1 (0–0.87) n = 1588	Q2 (0.87–2.17) n = 2076	Q3 (2.17–4.75) n = 2086	Q4 (4.75–254) n = 2183	p-value
Age, mean (SE)	54.4 (0.42)	57.2 (0.40)	57.0 (0.37)	57.2 (0.41)	<0.0001
Sex [No. (%)]					<0.0001
Male	917 (52.8 %)	1186 (54.9 %)	1033 (48.2 %)	869 (36.1 %)	
Female	671 (47.2 %)	890 (45.1 %)	1053 (51.8 %)	1314 (63.9 %)	
Race/Ethnicity [No. (%)]					<0.0001
White, Non-Hispanic	979 (82.0 %)	1218 (79.0 %)	1216 (79.6 %)	1131 (74.1 %)	
Black, Non-Hispanic	258 (7.0 %)	323 (7.8 %)	370 (8.8 %)	544 (13.3 %)	
Mexican American	247 (4.0 %)	404 (5.2 %)	386 (4.6 %)	401 (5.5 %)	
Other Race	104 (7.0 %)	131 (8.0 %)	114 (7.1 %)	107 (7.2 %)	
BMI (kg/m^2^)					<0.0001
<25	767 (51.7 %)	604 (28.3 %)	451 (20.5 %)	341 (15.3 %)	
25-30	603 (34.8 %)	936 (45.6 %)	821 (37.8 %)	645 (27.9 %)	
≥30	218 (13.5 %)	536 (26.1 %)	814 (41.7 %)	1197 (56.8 %)	
Education level [No. (%)]					<0.0001
High school graduate or below	746 (37.4 %)	1108 (41.8 %)	1141 (44.6 %)	1290 (50.7 %)	
Some college or above	842 (62.6 %)	968 (58.2 %)	945 (55.4 %)	893 (49.3 %)	
Cigarette smoking [No. (%)]					<0.0001
Never smoker	784 (50.2 %)	982 (48.1 %)	929 (44.3 %)	981 (45.3 %)	
Former smoker	536 (33.2 %)	739 (33.0 %)	717 (33.5 %)	711 (29.6 %)	
Current smoker	268 (16.6 %)	355 (18.9 %)	440 (22.2 %)	491 (25.1 %)	
Alcohol intake [No. (%)]					<0.0001
Never drinker	187 (9.7 %)	283 (11.8 %)	284 (11.3 %)	387 (14.8 %)	
Former drinker	239 (13.9 %)	350 (15.0 %)	404 (19.9 %)	477 (21.8 %)	
Current drinker	1162 (76.4 %)	1443 (73.2 %)	1398 (68.9 %)	1319 (63.4 %)	
Cancer [No. (%)]	195 (11.4 %)	257 (11.6 %)	258 (12.3 %)	314 (14.6 %)	0.019
Hypertension [No. (%)]	526 (29.2 %)	846 (36.1 %)	966 (42.0 %)	1168 (50.3 %)	<0.0001
Diabetes [No. (%)]	161 (7.0 %)	277 (9.0 %)	298 (11.0 %)	409 (15.6 %)	<0.0001
Heart failure [No. (%)]	49 (2.2 %)	84 (2.5 %)	94 (3.6 %)	156 (6.0 %)	<0.0001
Coronary heart disease [No. (%)]	97 (4.6 %)	144 (5.3 %)	130 (5.7 %)	176 (6.9 %)	0.054
Angina pectoris [No. (%)]	70 (3.5 %)	104 (3.9 %)	104 (3.9 %)	147 (6.4 %)	<0.0001
Myocardial infarction [No. (%)]	89 (4.3 %)	121 (4.5 %)	144 (5.4 %)	189 (7.3 %)	<0.0001
Stroke [No. (%)]	60 (2.4 %)	91 (2.6 %)	96 (3.9 %)	146 (5.5 %)	<0.0001
All-cause mortality [No. (%)]	439 (18.0 %)	704 (23.5 %)	789 (28.0 %)	881 (31.2 %)	<0.0001
Cancer mortality [No. (%)]	98 (5.1 %)	143 (6.3 %)	181 (8.2 %)	186 (9.3 %)	<0.0001
Cardiovascular mortality [No. (%)]	135 (6.2 %)	265 (9.8 %)	241 (10.1 %)	276 (12.1 %)	<0.0001
Follow-up time (months), mean (SE)	176.1 (1.70)	171.3 (1.24)	168.8 (1.65)	161.1 (1.26)	<0.0001
WBC count (1000 cells/μL), mean (SE)	6.25 (0.05)	6.59 (0.06)	7.03 (0.05)	7.69 (0.06)	<0.0001
Serum micronutrients					
Vitamin C (μmol/L) [mean (SE)]	54.00 (1.67)	48.43 (1.59)	42.92 (1.10)	38.32 (1.05)	<0.0001
25(OH)D (ng/mL) [mean (SE)]	61.92 (0.83)	59.51 (0.98)	57.89 (0.89)	52.71 (0.88)	<0.0001
α-tocopherol (μg/dL) [mean (SE)]	1325 (17.1)	1400 (17.8)	1389 (18.9)	1352 (14.8)	0.0013
β-carotene (μg/dL) [mean (SE)]	18.89 (0.63)	15.13 (0.50)	12.40 (0.38)	10.11 (0.31)	<0.0001
Lycopene (μg/dL) [mean (SE)]	20.62 (0.37)	19.83 (0.34)	18.71 (0.40)	17.11 (0.31)	<0.0001
Folate (ng/mL) [mean (SE)]	13.45 (0.23)	13.43 (0.24)	13.32 (0.20)	12.31 (0.25)	<0.0001
Iron (μmol/L) [mean (SE)]	16.02 (0.21)	15.23 (0.17)	14.59 (0.14)	11.83 (0.15)	<0.0001

SE: standard error; WBC: White blood cell; Q: quartile.

### 3.2 Associations of serum micronutrients with inflammatory biomarkers

Serum levels of all micronutrients, except α-tocopherol, were inversely correlated with serum CRP levels and WBC count (all p ≤ 0.001), with the correlations being appreciably stronger for serum CRP (Supplementary Table 1). After adjustment for confounders, serum levels of all micronutrients considered, except α-tocopherol, were linearly and inversely associated with serum CRP levels (all p ≤ 0.0025). A similar inverse association was also observed for serum vitamin C, β-carotene, and iron with WBC count (all p ≤ 0.0078) (Table 2).

**Table 2:** Multiple linear regression analysis of the associations between serum micronutrients and inflammatory biomarkers among participants in the National Health and Nutrition Examination Survey in 2001–2006 and 2017–2018.

Serum micronutrients^a^	Serum C-reactive protein (mg/L)		White blood cell count (1000 cells/μL)	
	Crude Model	Adjusted Model^b^	Crude Model	Adjusted Model^b^
Vitamin C (μmol/L)	n = 5186		n = 8356	
β (95 % CI)	**−0.29 (-0.38, -0.21)**	**−0.24 (-0.31, -0.17)**	**−0.051 (-0.065, -0.037)**	**−0.019 (-0.033, -0.0052)**
p	< **0.0001**	< **0.0001**	< **0.0001**	**0.0078**
25(OH)D (ng/mL)	n = 7891		n = 11,092	
β (95 % CI)	**−0.53 (-0.62, -0.44)**	**−0.16 (-0.26, -0.058)**	**−0.034 (-0.055, -0.014)**	−0.020 (−0.041, 0.0007)
p	< **0.0001**	**0.0025**	**0.0011**	0.058
α-tocopherol (μg/dL)	n = 7872		n = 11,002	
β (95 % CI)	0.024 (−0.064, 0.11)	0.064 (−0.015, 0.14)	−0.0001 (−0.021, 0.021)	**0.023 (0.0023, 0.043)**
p	0.59	0.11	0.99	**0.030**
β-carotene (μg/dL)	n = 7868		n = 10,947	
β (95 % CI)	**−0.37 (-0.42, -0.33)**	**−0.29 (-0.33, -0.25)**	**−0.069 (-0.078, -0.060)**	**−0.037 (-0.046, -0.028)**
p	< **0.0001**	< **0.0001**	< **0.0001**	< **0.0001**
Lycopene (μg/dL)	n = 7865		n = 10,904	
β (95 % CI)	**−0.24 (-0.30, -0.18)**	**−0.14 (-0.19, -0.08)**	**−0.022 (-0.036, -0.0087)**	−0.010 (−0.022, 0.0025)
p	< **0.0001**	< **0.0001**	**0.0017**	0.12
Folate (ng/mL)	n = 7903		n = 9687	
β (95 % CI)	**−0.16 (-0.22, -0.095)**	**−0.13 (-0.19, -0.069)**	**−0.031 (-0.044, -0.017)**	−0.0078 (−0.022, 0.0061)
p	< **0.0001**	< **0.0001**	< **0.0001**	0.27
Iron (μmol/L)	n = 7875		n = 11,022	
β (95 % CI)	**−0.79 (-0.89, -0.69)**	**−0.63 (-0.72, -0.54)**	**−0.10 (-0.12, -0.087)**	**−0.10 (-0.12, -0.086)**
p	< **0.0001**	< **0.0001**	< **0.0001**	< **0.0001**

CI: confidence interval.

a All variables were log-transformed.

b Adjustment for age, sex, race, body mass index, education, cigarette smoking, alcohol intake, cancer, hypertension, diabetes, heart failure, coronary heart disease, angina pectoris, myocardial infarction, and stroke.

Nonlinear dose-response associations between serum micronutrients and inflammatory biomarkers are shown in Supplementary Figs. 2 and 3. Specifically, we found L-shaped associations of serum 25(OH)D (p_nonlinearity_ = 0.0028) and serum folate (p_nonlinearity_ < 0.0001) with CRP **(**Supplementary Fig. 2B and 2F), reversed J-shaped associations of serum vitamin C and β-carotene with WBC count (Supplementary Fig. 3A and 3D) (all p_nonlinearity_ < 0.0001), and a U-shaped association between serum folate and WBC count (Supplementary Fig. 3F) (P_nonlinearity_ 0.0004).

After multivariate adjustment, the risk of persons with serum CRP (>3 mg/L) was decreased overall across the increasing quartiles of serum vitamin C, β-carotene, lycopene, folate, and iron (all p-trend ≤0.0024) (Table 3). For example, ORs (95 % CIs) for quartiles (Q) 2–4 vs. Q1 of serum β-carotene were 0.65 (0.57, 0.73), 0.55 (0.46, 0.65), and 0.34 (0.28, 0.42), respectively (p-trend <0.0001).

**Table 3:** ORs (95 % CIs) for high concentrations of serum C-reactive protein (>3 mg/L) in relation to serum micronutrients among participants in the National Health and Nutrition Examination Survey in 2001–2006.

Serum micronutrients	Quartile of Serum Micronutrients				p*-*trend
	1	2	3	4	
Vitamin C (μmol/L)					
Persons with CRP ≥/<3 mg/L	654/629	614/731	493/820	382/863	
Concentrations (median)	18.2	47.1	63.5	87.5	
Crude OR (95 % CI)	1.00	**0.79 (0.64, 0.97)**	**0.53 (0.44, 0.63)**	**0.38 (0.31, 0.48)**	< **0.0001**
Adjusted OR (95 % CI)^a^	1.00	0.81 (0.65, 1.01)	**0.61 (0.50, 0.75)**	**0.43 (0.34, 0.55)**	< **0.0001**
25(OH)D (ng/mL)					
Persons with CRP ≥/<3 mg/L	1303/1277	770/1210	662/1118	527/1024	
Concentrations (median)	36.7	54.1	67	85.1	
Crude OR (95 % CI)	1.00	**0.64 (0.53, 0.77)**	**0.58 (0.49, 0.69)**	**0.48 (0.39, 0.59)**	< **0.0001**
Adjusted OR (95 % CI)^a^	1.00	**0.82 (0.67, 0.99)**	0.84 (0.70, 1.02)	0.84 (0.65, 1.07)	0.17
α-tocopherol (μg/dL)					
Persons with CRP ≥/<3 mg/L	891/1179	835/1112	778/1153	748/1176	
Concentrations (median)	905	1179.7	1480.4	2159.5	
Crude OR (95 % CI)	1.00	0.94 (0.83, 1.08)	0.91 (0.77, 1.07)	0.87 (0.76, 1.01)	0.0688
Adjusted OR (95 % CI)^a^	1.00	1.05 (0.91, 1.20)	1.05 (0.86, 1.27)	0.99 (0.83, 1.19)	0.95
β-carotene (μg/dL)					
Persons with CRP ≥/<3 mg/L	1038/861	837/1060	784/1262	590/1436	
Concentrations (median)	5.1	10.2	17.9	37.4	
Crude OR (95 % CI)	1.00	**0.62 (0.55, 0.70)**	**0.50 (0.42, 0.58)**	**0.28 (0.23, 0.33)**	< **0.0001**
Adjusted OR (95 % CI)^a^	1.00	**0.65 (0.57, 0.73)**	**0.55 (0.46, 0.65)**	**0.34 (0.28, 0.42)**	< **0.0001**
Lycopene (μg/dL)					
Persons with CRP ≥/<3 mg/L	1132/1280	849/1197	730/1072	537/1068	
Concentrations (median)	9.9	17.3	24.4	35.2	
Crude OR (95 % CI)	1.00	**0.74 (0.62, 0.88)**	**0.72 (0.60, 0.85)**	**0.51 (0.41, 0.64)**	< **0.0001**
Adjusted OR (95 % CI)^a^	1.00	**0.77 (0.63, 0.93)**	**0.79 (0.65, 0.95)**	**0.64 (0.51, 0.82)**	**0.0005**
Folate (ng/mL)					
Persons with CRP ≥/<3 mg/L	997/1067	841/1155	674/1188	754/1227	
Concentrations (median)	7.2	11.1	15.3	23.7	
Crude OR (95 % CI)	1.00	**0.80 (0.68, 0.93)**	**0.64 (0.55, 0.74)**	**0.70 (0.58, 0.83)**	< **0.0001**
Adjusted OR (95 % CI)^a^	1.00	**0.85 (0.74, 0.98)**	**0.72 (0.62, 0.84)**	**0.77 (0.63, 0.94)**	**0.0024**
Iron (μmol/L)					
Persons with CRP ≥/<3 mg/L	1239/818	870/1168	670/1300	478/1332	
Concentrations (median)	9	12.9	16.7	22.4	
Crude OR (95 % CI)	1.00	**0.53 (0.46, 0.61)**	**0.36 (0.31, 0.42)**	**0.26 (0.22, 0.32)**	< **0.0001**
Adjusted OR (95 % CI)^a^	1.00	**0.53 (0.45, 0.62)**	**0.39 (0.33, 0.46)**	**0.32 (0.27, 0.39)**	< **0.0001**

OR: odds ratio; CI: confidence interval; CRP: C-reactive protein.

a Adjustment for age, sex, race, body mass index, education, cigarette smoking, alcohol intake, cancer, hypertension, diabetes, heart failure, coronary heart disease, angina pectoris, myocardial infarction, and stroke.

### 3.3 Associations of serum micronutrients and inflammatory biomarkers with all-cause, cancer, and cardiovascular mortality

The risk of death from all causes, cancer, and CVD was decreasing across increasing quartiles of serum vitamin C, 25(OH)D, β-carotene, and lycopene (p-trend: 0.026 - <0.0001) (Table 4). For example, multivariable-adjusted HRs (95 % CIs) for cancer mortality, comparing Qs 2–4 with Q1 of serum vitamin C, were 0.68 (0.48, 0.96), 0.49 (0.38, 0.64), and 0.45 (0.33, 0.62) (p-trend: <0.0001), respectively. Corresponding HRs (95 % CIs) for cardiovascular mortality were 0.71 (0.48, 1.04), 0.63 (0.45, 0.89), and 0.59 (0.44, 0.79) (p-trend: 0.0008) (Table 4). Conversely, serum CRP was positively associated with all-cause, cancer, and cardiovascular mortality (all p-trend across quartiles: ≤0.01). Compared with subjects in the first (i.e., lowest) quartile of serum CRP, those in the fourth quartile exhibited a 57 % and 63 % elevated risk of cancer mortality and cardiovascular mortality, respectively (Table 4).

**Table 4:** The associations of serum micronutrients and inflammatory biomarkers with all-cause, cancer, and cardiovascular mortality among participants in the National Health and Nutrition Examination Survey in 2001–2006 and 2017–2018.

Variables	All-cause mortality (n = 11,539)		Cancer mortality (n = 9183)		Cardiovascular mortality (n = 9488)	
	Crude Model HR (95 % CI)	Adjusted Model^a^ HR (95 % CI)	Crude Model HR (95 % CI)	Adjusted Model^a^ HR (95 % CI)	Crude Model HR (95 % CI)	Adjusted Model^a^ HR (95 % CI)
Vitamin C (μmol/L)						
Q1	1.00	1.00	1.00	1.00	1.00	1.00
Q2	**0.76 (0.63, 0.92)**	**0.73 (0.60, 0.90)**	**0.70 (0.50, 0.97)**	**0.68 (0.48, 0.96)**	0.88 (0.61, 1.25)	0.71 (0.48, 1.04)
Q3	**0.71 (0.61, 0.82)**	**0.61 (0.52, 0.72)**	**0.57 (0.44, 0.76)**	**0.49 (0.38, 0.64)**	0.87 (0.66, 1.16)	**0.63 (0.45, 0.89)**
Q4	0.97 (0.82, 1.15)	**0.64 (0.53, 0.78)**	**0.69 (0.51, 0.93)**	**0.45 (0.33, 0.62)**	1.09 (0.84, 1.41)	**0.59 (0.44, 0.79)**
p-trend	0.73	< **0.0001**	**0.012**	< **0.0001**	0.50	**0.0008**
25(OH)D (ng/mL)						
Q1	1.00	1.00	1.00	1.00	1.00	1.00
Q2	**0.73 (0.65, 0.83)**	**0.72 (0.62, 0.83)**	**0.72 (0.56, 0.91)**	**0.68 (0.52, 0.91)**	**0.64 (0.52, 0.78)**	**0.64 (0.50, 0.83)**
Q3	**0.71 (0.62, 0.80)**	**0.70 (0.62, 0.79)**	**0.57 (0.42, 0.79)**	**0.54 (0.39, 0.73)**	**0.71 (0.59, 0.85)**	**0.66 (0.53, 0.83)**
Q4	**0.63 (0.52, 0.76)**	**0.66 (0.56, 0.78)**	**0.52 (0.36, 0.76)**	**0.48 (0.32, 0.71)**	**0.54 (0.40, 0.73)**	**0.59 (0.42, 0.82)**
p-trend	< **0.0001**	< **0.0001**	**0.0002**	**0.0001**	< **0.0001**	**0.0012**
α-tocopherol (μg/dL)						
Q1	1.00	1.00	1.00	1.00	1.00	1.00
Q2	**0.86 (0.75, 0.99)**	0.90 (0.78, 1.04)	**0.75 (0.58, 0.98)**	**0.74 (0.57, 0.96)**	**0.79 (0.64, 0.97)**	0.81 (0.64, 1.02)
Q3	1.06 (0.92, 1.22)	0.90 (0.78, 1.05)	0.91 (0.67, 1.23)	0.82 (0.59, 1.15)	0.92 (0.75, 1.12)	0.78 (0.60, 1.02)
Q4	1.25 (1.12, 1.38)	**0.83 (0.70, 0.98)**	1.07 (0.87, 1.31)	0.75 (0.55, 1.02)	1.27 (1.03, 1.56)	0.73 (0.53, 1.03)
p-trend	<0.0001	**0.040**	0.33	0.15	0.016	0.096
β-carotene (μg/dL)						
Q1	1.00	1.00	1.00	1.00	1.00	1.00
Q2	0.90 (0.79, 1.03)	**0.79 (0.67, 0.93)**	0.89 (0.70, 1.14)	0.87 (0.66, 1.15)	0.98 (0.76, 1.26)	0.79 (0.56, 1.11)
Q3	0.91 (0.79, 1.05)	**0.71 (0.61, 0.83)**	0.83 (0.67, 1.03)	0.78 (0.61, 1.00)	0.99 (0.76, 1.27)	**0.68 (0.51, 0.92)**
Q4	0.93 (0.80, 1.10)	**0.68 (0.58, 0.80)**	0.82 (0.63, 1.07)	**0.73 (0.54, 0.99)**	0.95 (0.75, 1.21)	**0.59 (0.44, 0.80)**
p-trend	0.445	< **0.0001**	0.092	**0.026**	0.71	**0.0002**
Lycopene (μg/dL)						
Q1	1.00	1.00	1.00	1.00	1.00	1.00
Q2	**0.56 (0.49, 0.63)**	**0.85 (0.76, 0.94)**	**0.69 (0.55, 0.86)**	0.99 (0.81, 1.20)	**0.47 (0.39, 0.56)**	**0.76 (0.65, 0.89)**
Q3	**0.46 (0.40, 0.53)**	**0.83 (0.72, 0.95)**	**0.43 (0.32, 0.58)**	**0.71 (0.52, 0.95)**	**0.39 (0.32, 0.48)**	**0.73 (0.58, 0.93)**
Q4	**0.26 (0.22, 0.31)**	**0.65 (0.56, 0.75)**	**0.27 (0.19, 0.39)**	**0.56 (0.37, 0.83)**	**0.22 (0.16, 0.31)**	**0.64 (0.48, 0.86)**
p-trend	< **0.0001**	< **0.0001**	< **0.0001**	**0.0007**	< **0.0001**	**0.001**
Folate (ng/mL)						
Q1	1.00	1.00	1.00	1.00	1.00	1.00
Q2	0.95 (0.79, 1.14)	0.90 (0.76, 1.05)	0.93 (0.68, 1.27)	0.89 (0.64, 1.23)	0.93 (0.71, 1.24)	0.79 (0.60, 1.05)
Q3	1.09 (0.95, 1.24)	**0.87 (0.77, 0.98)**	0.87 (0.65, 1.16)	0.81 (0.61, 1.08)	1.22 (0.99, 1.50)	0.83 (0.66, 1.06)
Q4	1.87 (1.63, 2.14)	0.91 (0.78, 1.06)	1.64 (1.25, 2.15)	0.92 (0.64, 1.31)	2.18 (1.78, 2.68)	0.83 (0.63, 1.11)
p-trend	<0.0001	0.22	0.0026	0.59	<0.0001	0.34
Iron (μmol/L)						
Q1	1.00	1.00	1.00	1.00	1.00	1.00
Q2	**0.88 (0.79, 0.98)**	**0.85 (0.75, 0.97)**	0.86 (0.65, 1.14)	0.78 (0.57, 1.07)	0.83 (0.66, 1.03)	0.89 (0.71, 1.10)
Q3	**0.86 (0.76, 0.97)**	**0.83 (0.73, 0.95)**	1.00 (0.74, 1.36)	0.91 (0.69, 1.20)	0.84 (0.67, 1.04)	0.84 (0.67, 1.04)
Q4	**0.73 (0.65, 0.83)**	**0.81 (0.73, 0.91)**	0.91 (0.69, 1.18)	0.89 (0.68, 1.18)	**0.64 (0.50, 0.82)**	**0.76 (0.59, 0.96)**
p-trend	< **0.0001**	**0.0003**	0.74	0.66	**0.0009**	**0.018**
C-reactive protein (mg/L)						
Q1	1.00	1.00	1.00	1.00	1.00	1.00
Q2	**1.35 (1.15, 1.59)**	1.08 (0.92, 1.26)	1.27 (0.87, 1.85)	1.04 (0.72, 1.51)	**1.62 (1.30, 2.01)**	1.25 (0.99, 1.57)
Q3	**1.64 (1.39, 1.92)**	**1.34 (1.14, 1.58)**	**1.65 (1.18, 2.31)**	**1.35 (0.95, 1.93)**	**1.67 (1.33, 2.10)**	**1.37 (1.06, 1.78)**
Q4	**1.92 (1.62, 2.29)**	**1.60 (1.34, 1.91)**	**1.81 (1.27, 2.59)**	**1.57 (1.08, 2.27)**	**1.96 (1.53, 2.51)**	**1.63 (1.23, 2.18)**
p-trend	< **0.0001**	< **0.0001**	**0.0009**	**0.010**	< **0.0001**	**0.0017**
WBC count (1000 cells/μL)						
Q1	1.00	1.00	1.00	1.00	1.00	1.00
Q2	**1.23 (1.07, 1.43)**	1.08 (0.95, 1.22)	**1.39 (1.03, 1.88)**	1.29 (0.96, 1.74)	1.05 (0.80, 1.38)	0.89 (0.69, 1.14)
Q3	**1.17 (1.00, 1.37)**	1.01 (0.88, 1.16)	**1.39 (1.06, 1.82)**	1.25 (0.96, 1.63)	1.20 (0.92, 1.57)	0.98 (0.79, 1.22)
Q4	**1.38 (1.19, 1.61)**	**1.23 (1.10, 1.38)**	**1.59 (1.14, 2.21)**	**1.52 (1.13, 2.04)**	1.21 (0.88, 1.65)	1.17 (0.90, 1.52)
p-trend	**0.0004**	**0.0098**	**0.0058**	**0.0057**	0.14	0.17

HR: hazard ratio; CI: confidence interval; WBC: white blood cell; Q: quartile.

a Adjustment for age, sex, race, body mass index, education, cigarette smoking, alcohol intake, cancer, hypertension, diabetes, heart failure, coronary heart disease, angina pectoris, myocardial infarction, and stroke.

**Fig. 1:**
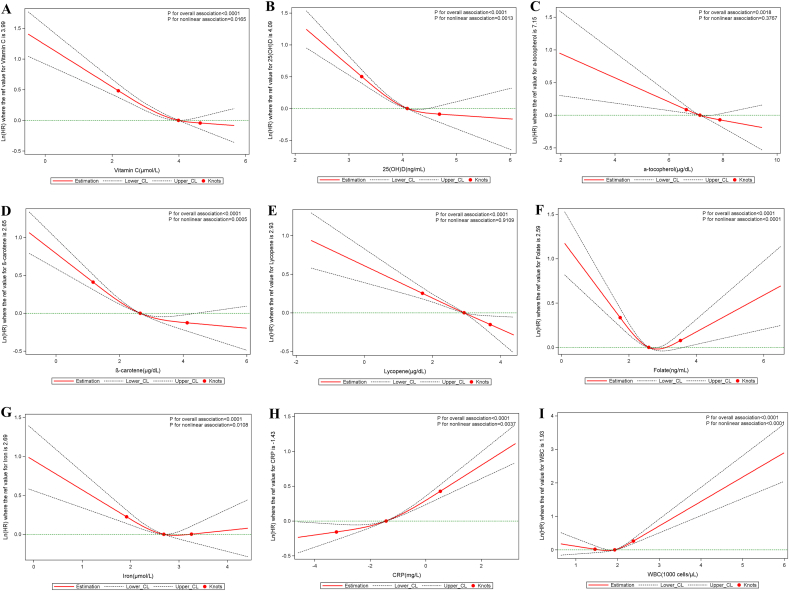
Nonlinear dose-response associations of serum micronutrients and inflammatory biomarkers with all-cause mortality. All serum micronutrients and inflammatory biomarkers were log-transformed and modeled using restricted cubic spline function with three knots located at the 5th, 50th, and 95th percentiles. Y represents the natural logarithm of the hazard ratio (Ln(HR)) for serum micronutrients or inflammatory biomarkers, relative to their median values, which serve as the reference. Knots are indicated by dots. The model was adjusted for age, sex, race, body mass index, education, cigarette smoking, alcohol intake, cancer, hypertension, diabetes, heart failure, coronary heart disease, angina pectoris, myocardial infarction, and stroke.

**Fig. 2:**
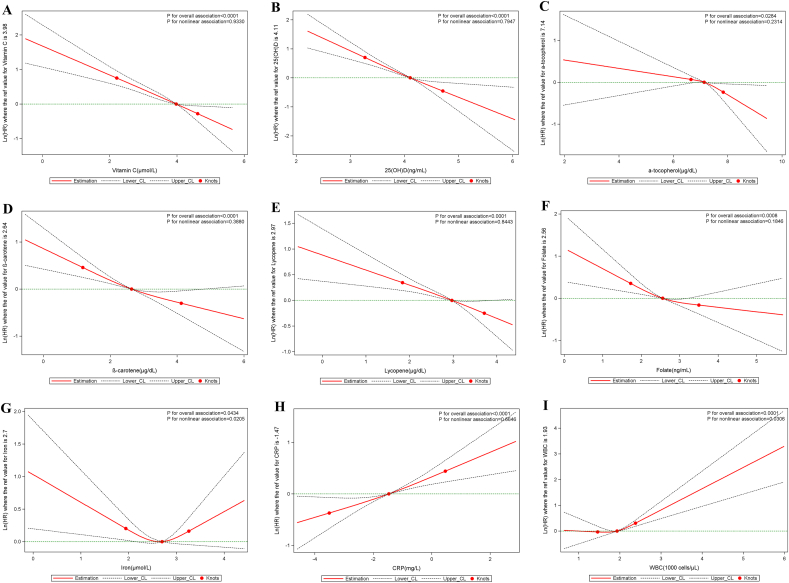
Nonlinear dose-response associations of serum micronutrients and inflammatory biomarkers with cancer mortality. All serum micronutrients and inflammatory biomarkers were log-transformed and modeled using restricted cubic spline function with three knots located at the 5th, 50th, and 95th percentiles. Y represents the natural logarithm of the hazard ratio (Ln(HR)) for serum micronutrients or inflammatory biomarkers, relative to their median values, which serve as the reference. Knots are indicated by dots. The model was adjusted for age, sex, race, body mass index, education, cigarette smoking, alcohol intake, cancer, hypertension, diabetes, heart failure, coronary heart disease, angina pectoris, myocardial infarction, and stroke.

**Fig. 3:**
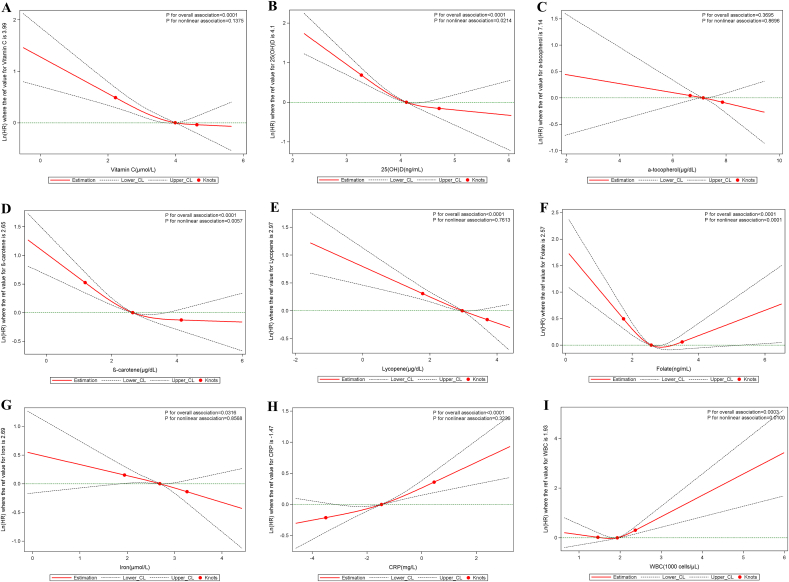
Nonlinear dose-response associations of serum micronutrients and inflammatory biomarkers with cardiovascular mortality. All serum micronutrients and inflammatory biomarkers were log-transformed and modeled using restricted cubic spline function with three knots located at the 5th, 50th, and 95th percentiles. Y represents the natural logarithm of the hazard ratio (Ln(HR)) for serum micronutrients or inflammatory biomarkers, relative to their median values, which serve as the reference. Knots are indicated by dots. The model was adjusted for age, sex, race, body mass index, education, cigarette smoking, alcohol intake, cancer, hypertension, diabetes, heart failure, coronary heart disease, angina pectoris, myocardial infarction, and stroke.

L-shaped associations were found between serum vitamin C, 25(OH)D, β-carotene, and iron and all-cause mortality (all p_nonlinearity_ < 0.05), between serum 25(OH)D and β-carotene and cardiovascular mortality (all p_nonlinearity_ < 0.022), and between serum iron and cancer mortality (p_nonlinearity_ < 0.021). A U-shaped association appeared between serum folate and all-cause and cardiovascular mortality (p_nonlinearity_ < 0.0001). The associations between serum CRP and all-cause mortality (p_nonlinearity_ < 0.0001) and the associations between WBC count and the three mortality outcomes considered existed in a non-linear manner (p_nonlinearity_ < 0.05). All non-linear associations described above were independent of established and suspected confounders (Fig. 1, Fig. 2, Fig. 3**)**.

### 3.4 Mediating role of inflammatory biomarkers

Serum CRP significantly mediated the associations of vitamin C, 25(OH)D, β-carotene, lycopene, folate, and iron with all-cause, cancer, and cardiovascular mortality, except for the association between iron and cancer mortality. Serum C-reactive protein significantly mediated 5.3%–20.4 %, 4.5%–18.1 %, and 3.3%–15.7 % of the associations of vitamin C, 25(OH)D, β-carotene, and lycopene with all-cause, cancer, and cardiovascular mortality, respectively (Table 5). The mediation effects of WBC count on the associations of vitamin C, β-carotene, lycopene, and iron with all-cause, cancer, and cardiovascular mortality were overall weaker than the corresponding mediation effects of serum CRP **(**Supplementary Table 2).

**Table 5:** Mediation analysis of C-reactive protein for the associations between serum micronutrients and all-cause mortality, cancer mortality, and cardiovascular mortality among participants in the National Health and Nutrition Examination Survey in 2001–2006^a^.

Serum micronutrients	Total effect		Indirect effect		Direct effect		Proportion Mediated^b^ (%)
	Coefficients (95%CI)	p-value	Coefficients (95%CI)	p-value	Coefficients (95%CI)	p-value	
All-cause mortality							
Vitamin C	58.41 (48.48, 68.26)	<0.0001	10.28 (7.25, 13.85)	<0.0001	48.13 (37.31, 57.93)	<0.0001	**17.5**
25(OH)D	62.97 (58.80, 67.39)	<0.0001	3.40 (1.79, 5.36)	<0.0001	59.57 (54.81, 64.26)	<0.0001	**5.3**
α-tocopherol	28.56 (17.63, 33.93)	<0.0001	−1.37 (−3.73, 0.56)	0.18	29.93 (18.76, 35.25)	<0.0001	NA
β-carotene	54.11 (42.03, 66.03)	<0.0001	11.04 (7.94, 14.40)	<0.0001	43.07 (31.25, 54.90)	<0.0001	**20.4**
Lycopene	55.99 (44.49, 66.96)	<0.0001	6.16 (3.95, 8.60)	<0.0001	49.84 (37.79, 60.67)	<0.0001	**10.9**
Folate	35.81 (12.32, 55.04)	0.002	7.35 (4.53, 10.70)	<0.0001	28.46 (5.27, 47.98)	0.016	**20.1**
Iron	51.56 (29.11, 70.97)	<0.0001	31.38 (22.50, 42.07)	<0.0001	20.18 (−7.77, 43.82)	0.14	**59.8**
Cancer mortality							
Vitamin C	348.00 (247.58, 484.36)	<0.0001	51.38 (22.85, 90.83)	<0.0001	296.62 (207.71, 410.90)	<0.0001	**14.4**
25(OH)D	228.40 (152.65, 317.48)	<0.0001	10.94 (3.93, 22.76)	<0.0001	217.46 (146.99, 298.30)	<0.0001	**4.5**
α-tocopherol	156.36 (106.77, 212.17)	0.002	−7.19 (−27.66, 3.95)	0.24	163.55 (112.27, 226.90)	0.002	NA
β-carotene	414.63 (262.87, 592.88)	<0.0001	76.71 (39.54, 128.30)	<0.0001	337.92 (188.85, 498.99)	<0.0001	**18.1**
Lycopene	393.25 (274.21, 520.35)	<0.0001	33.35 (15.01, 59.77)	<0.0001	359.90 (242.13, 483.38)	<0.0001	**8.1**
Folate	434.55 (218.47, 627.64)	<0.0001	65.86 (32.60, 110.60)	<0.0001	368.69 (148.80, 561.72)	0.006	**14.7**
Iron	82.28 (−489.66, 443.24)	0.60	301.19 (143.00, 542.73)	<0.0001	−218.91 (−985.31, 233.12)	0.53	84.0
Cardiovascular mortality							
Vitamin C	257.45 (177.88, 338.16)	<0.0001	36.66 (18.20, 63.01)	<0.0001	220.79 (140.83, 300.36)	<0.0001	**14.0**
25(OH)D	223.93 (177.47, 278.49)	<0.0001	7.90 (2.65, 16.60)	<0.0001	216.03 (171.74, 266.74)	<0.0001	**3.3**
α-tocopherol	88.58 (−32.50, 146.05)	0.096	−12.71 (−31.82, −1.83)	0.026	101.30 (−14.69, 156.37)	0.062	NA
β-carotene	251.68 (168.71, 352.26)	<0.0001	40.07 (20.58, 62.91)	<0.0001	211.60 (128.11, 302.74)	0.002	**15.7**
Lycopene	250.92 (175.90, 325.39)	<0.0001	20.98 (9.97, 35.47)	<0.0001	229.94 (155.82, 300.36)	<0.0001	**8.2**
Folate	191.40 (29.92, 320.16)	0.022	30.99 (15.66, 52.00)	<0.0001	160.41 (−9.08, 290.38)	0.064	**15.1**
Iron	229.99 (66.37, 360.27)	0.006	96.93 (49.80, 164.00)	<0.0001	133.06 (−62.44, 279.38)	0.14	**39.2**

CI: confidence interval.

a All serum micronutrients and C-reactive protein were log-transformed. Causal mediation analysis was performed by adjusting for age, sex, race, body mass index, education, cigarette smoking, alcohol intake, cancer, hypertension, diabetes, heart failure, coronary heart disease, angina pectoris, myocardial infarction, and stroke. The coefficients represent the estimated change in the logarithm of survival time for each unit increase in serum micronutrients.

b NA: the proportion mediated was not computed when the point estimate of the direct effect was in the opposite direction to that of the indirect effect.

The results described above remained virtually unchanged after excluding participants with less than two years of follow-up.

## 4 Discussion

In this nationally representative sample of the U.S. population, we found that serum levels of vitamin C, 25(OH)D, β-carotene, and lycopene were inversely associated with all-cause, cancer, and cardiovascular mortality after adjustment for confounders. These associations were largely L-shaped and mediated to various extents by inflammatory biomarkers considered. The observed mediation effects are biologically plausible because serum CRP and WBC count were inversely associated with serum levels of most micronutrients examined and positively associated with all-cause, cancer, and/or cardiovascular mortality. These findings offer additional epidemiological and mechanistic evidence for the crucial roles of inflammation in the pathogenesis of cancer and CVD.

Some micronutrients (e.g., vitamin C, α-tocopherol, β-carotene, lycopene) are antioxidants that scavenge free radicals inside the cell and thus reduce oxidative damage and inflammation in the body [36]. We observed significantly inverse associations between vitamin C, 25(OH)D, β-carotene, lycopene, folate, and iron with serum CRP and WBC count, but a similar association was not identified for serum α-tocopherol. Our findings are consistent with those of some, but not all, previous studies [13,37]. Most studies revealed that individuals who took α-tocopherol as a supplement or were assigned to receive it in randomized trials had a reduced level of serum CRP compared with controls [38,39]. However, no association between serum α-tocopherol and CRP was observed in a cross-sectional study [13] and no effect of α-tocopherol supplementation on WBC count was reported in a randomized trial [40]. The discrepant results between the present study and some other studies on α-tocopherol are in part attributable to differences in the source and amount of exposure to this vitamin. In our study population, α-tocopherol was primarily derived from dietary intake, whereas it was administered in pharmacological doses to participants in most randomized trials [39].

We further investigated whether serum micronutrients are associated with serum inflammatory biomarkers in a non-linear manner. An L-shaped association between serum 25(OH)D and CRP was observed in our study. Serum CRP levels gradually decreased as 25(OH)D levels increased. This increase continued until serum 25(OH)D reached 56.8 ng/ml, after which CRP levels appeared to stabilize. This non-linear pattern of the association is consistent with the findings of a Mendelian randomization study [41]. Additionally, a cross-sectional study demonstrated an L-shaped association between serum 25(OH)D and CRP in individuals with metabolic diseases, whereas the association was linear in those with pulmonary, gastrointestinal and psychiatric diseases [42]. In contrast, an analysis of the UK Biobank data found no significant association between serum vitamin D and CRP levels [14]. To date, only one observational study has examined the non-linear associations between serum levels of other micronutrients with serum levels of inflammatory biomarkers [43]. That study identified an L-shaped association between serum vitamin C and high-sensitivity CRP [43]. However, in the present study with a larger sample size, the association between serum vitamin C and CRP appeared to be linear. The reasons for the observed inconsistent patterns of the associations described above are largely unclear but may be ascribed to differences in demographic and other health-related characteristics of study subjects and adjustment of confounders between previous studies [14].

It is warranted to understand the complex long-term effects of various micronutrients on all-cause and cause-specific mortality. The present study showed that elevated serum levels of vitamin C, 25(OH)D, β-carotene, and lycopene were associated with reduced all-cause, cancer, and cardiovascular mortality. Furthermore, the patterns of these associations varied by the micronutrients examined. Our findings are overall consistent with those of previous studies. A meta-analysis of prospective studies revealed that dietary intake and/or blood levels of vitamin C, β-carotene, lycopene, and α-tocopherol were inversely associated with all-cause, cancer, and cardiovascular mortality [17]. A reduced risk of death from cancer and cardiovascular mortality associated with circulating levels of 25(OH)D was also observed in a prospective cohort study [14].

The findings of the present study are inconsistent with those of most intervention studies examining the effects of micronutrient supplementation on mortality outcomes. Meta-analyses of randomized trials showed overall null effects of β-carotene supplementation on cancer and cardiovascular mortality [23] and vitamin E supplementation on all-cause mortality [44]. Most randomized trials found that vitamin D supplementation reduced all-cause mortality, but not cardiovascular mortality [45]. The discrepant results between observational studies (including our own) and randomized chemopreventive trials may be primarily attributable to confounding in the former and weaknesses of the latter. Some weaknesses in randomized trials include failure to capture usual exposure to micronutrients in early-stage of life, use of pharmacological doses of micronutrients, recruitment of high-risk populations with a relatively short period of follow-up, and unsatisfactory adherence of subjects to micronutrient supplementation [46].

A question arises as to whether a threshold effect exists for the associations between serum micronutrients of interest and risk of death from all causes, cancer, and cardiovascular disease. We observed L-shaped associations of serum vitamin C, 25(OH)D, β-carotene, and iron with all-cause, cancer, and/or cardiovascular mortality, suggesting that the protective effects of elevated serum levels of those micronutrients on all-cause and cause-specific mortality tend to plateau once their levels have reached certain thresholds. Serum folate exhibited a U-shaped association with all-cause and cardiovascular mortality and an L-shaped association with cancer mortality. Similarly, a study by Peng et al. reported that both extremely low and high serum levels of folate were associated with an increased risk of death from all causes, cancer, and CVD [47]. This risk pattern highlights the potential health hazards conferred by insufficient or excessive intake of folate. Experimental studies have elucidated that too much folate intake promoted tumorigenesis by increasing promoter methylation of tumor suppressor genes and exacerbated atherosclerosis by inducing aberrant DNA methylation [48].

It remains elusive how intake of micronutrients exerts a beneficial effect on cancer, CVD, and other chronic diseases. Inflammation is implicated in the initiation and progression of both cancer and CVD by modulating several signaling pathways, including vascular endothelial growth factor, nuclear factor-kappa B, and transforming growth factor beta [3]. Additionally, anti-inflammatory treatments favorably influenced cancer and CVD outcomes [49,50]. All the above experimental evidence prompted us to hypothesize that inflammation modulates the effects of micronutrients on cancer and cardiovascular mortality. The findings of the present study provided substantial supporting data for this hypothesis. For example, CRP mediated 5.3 %, 4.5 %, and 3.3 % of the association of serum 25(OH)D with all-cause, cancer, and cardiovascular mortality, respectively. We identified only one study that evaluated the mediation effects of inflammatory biomarkers on the associations between serum micronutrients and all-cause and cause-specific mortality [14]. In that study, serum CRP did not significantly mediate the association of vitamin D with all-cause and cause-specific mortality among participants in the UK Biobank cohort. The inconsistent results between the present study and the UK Biobank cohort study were obtained probably due to their differences in measurement methods for serum CRP, population characteristics, and confounding control.

The advantages of the present study include an analysis of data collected from a nationally representative population, the measurements of serum micronutrients and inflammation biomarkers using validated experimental methods, and consideration of two inflammation biomarkers (i.e., CRP and WBC count). In addition, we evaluated both linear and non-linear associations between serum micronutrients and all-cause and cause-specific mortality, which allows us to evaluate both strength and patterns of those associations. Specifically, our study offers novel evidence that serum vitamin C, 25(OH)D, β-carotene, and iron have a threshold effect on all-cause, cancer, and/or cardiovascular mortality. The present study is one of the first epidemiological studies to identify mediation factors for the potential beneficial effects of increased intake of micronutrients on the risk of death from cancer and CVD.

The present study has several limitations. First, the inverse associations between serum micronutrients and inflammatory biomarkers were examined in a cross-sectional study. It is possible that chronic diseases or inflammatory conditions might have influenced the serum levels of micronutrients considered. However, reverse causality is unlikely as lifestyle factors and several chronic diseases related to inflammation were adjusted in our data analysis. Second, serum levels of micronutrients measured only at baseline were evaluated in relation to all-cause, cancer, and cardiovascular mortality. One-time measurement was not able to capture changes in serum levels of micronutrients considered over time, which could lead to misclassification of individuals regarding their levels of micronutrient exposure. If such a misclassification is non-differential, it tends to bias our risk estimates toward the null. Third, a relatively small number of deaths from cancer in our study population makes it impossible for us to investigate the associations between serum micronutrients and site-specific cancer mortality.

In conclusion, the present study showed that higher serum levels of vitamin C, 25(OH)D, β-carotene, and lycopene were associated with a lower risk of death from all causes, cancer, and CVD in a large general U.S. population. Furthermore, these associations were mediated in part through inflammatory biomarkers, particularly serum CRP. Future studies should investigate the circulating levels and trajectories of serum micronutrients and inflammatory biomarkers in relation to the risk of cancer, CVD, and other chronic diseases in populations with diverse dietary habits and other lifestyle factors. Such research has tremendous public health impact as it is expected to provide innovative and practical approaches for preventing chronic diseases by dietary modifications.

## CRediT authorship contribution statement

**Chunliang Liu:** Conceptualization, Data curation, Formal analysis, Writing – original draft. **Harrison Wongsonegoro:** Conceptualization, Data curation, Formal analysis. **Tianchen Sheng:** Writing – review & editing. **Hao Fan:** Writing – review & editing. **Jianjun Zhang:** Conceptualization, Supervision, Writing – original draft.

## Ethics statement

The study protocols of NHANES were approved by the National Center for Health Statistic Ethics Review Board and written informed consent was obtained from all participants.

## Data availability

The NHANES data analyzed in the present study are available at: https://www.cdc.gov/nchs/nhanes/index.htm.

## Funding

No specific funding was received for this research.

## Declaration of competing interest

The authors declare that they have no known competing financial interests or personal relationships that could have appeared to influence the work reported in this paper.
