# Supplementary material for: Associations between serum micronutrients and all-cause, cancer, and cardiovascular mortality in a national representative population: Mediated by inflammatory biomarkers
Source: Redox Biol. 2025 Feb 26;81:103573. doi: 10.1016/j.redox.2025.103573 (PMC11915157; doi:10.1016/j.redox.2025.103573)
Supplement: Multimedia component 1 [file mmc1.pdf]

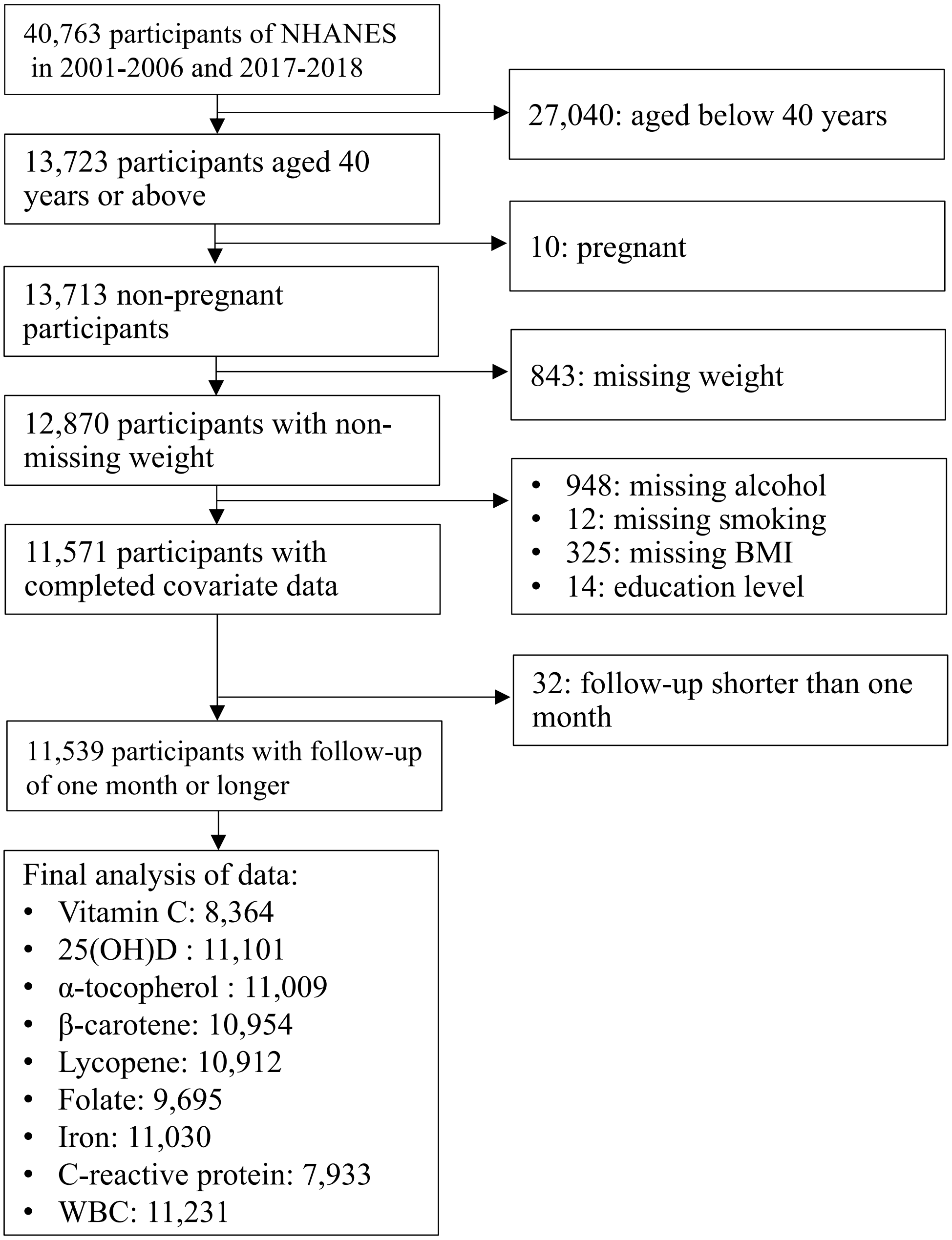

Supplementary Figure 1. Flow chart of study participants

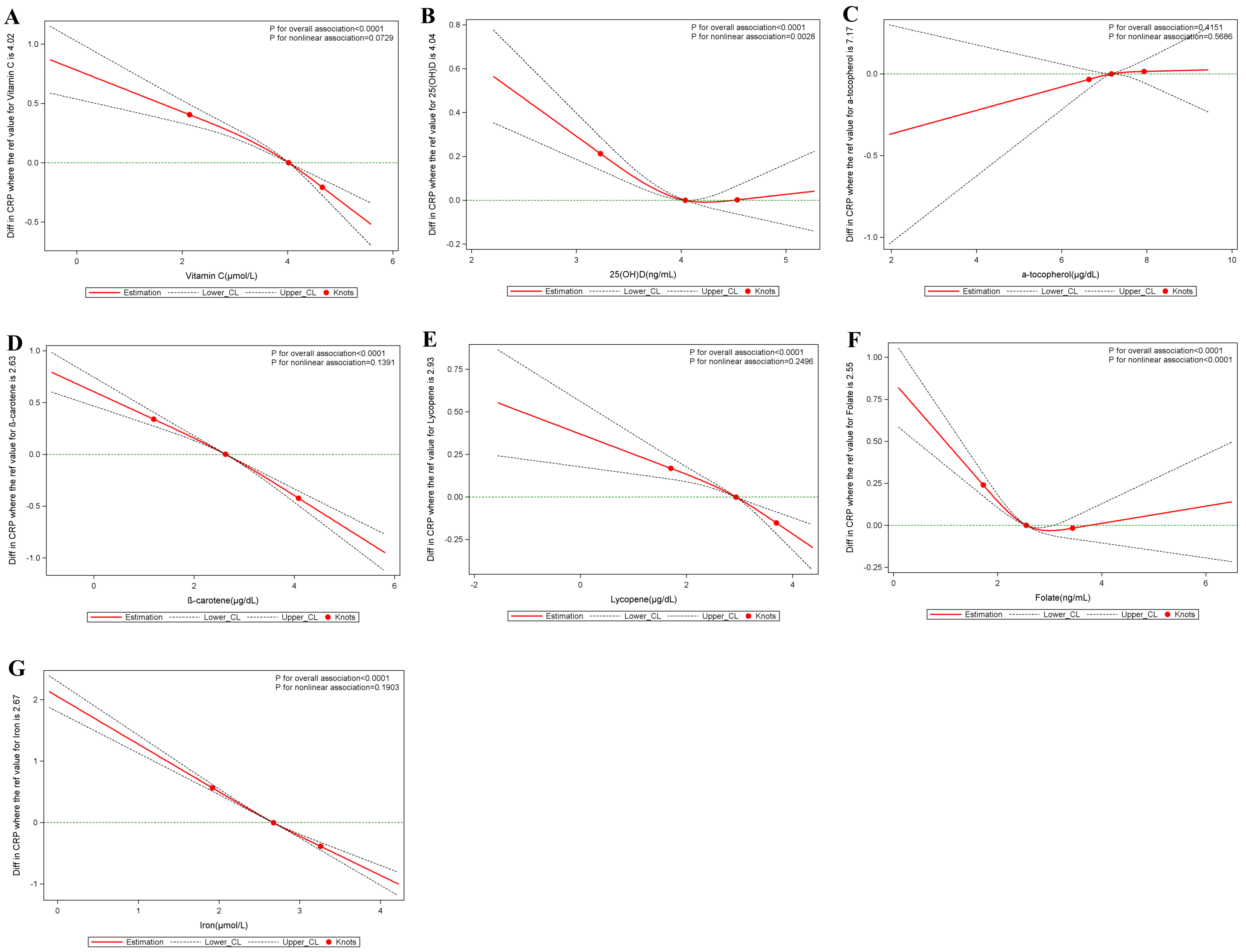

Supplementary Figure 2. Nonlinear dose-response associations between serum micronutrients and C-reactive protein (CRP). All variables were log-transformed. Serum micronutrients were modeled using restricted cubic spline function with three knots located at the 5th, 50th, and 95th percentiles. Y represents the difference (Diff) in CRP for serum micronutrients, relative to their median values, which serve as the reference. Knots are indicated by dots. The model was adjusted for age, sex, race, body mass index, education, cigarette smoking, alcohol intake, cancer, hypertension, diabetes, heart failure, coronary heart disease, angina pectoris, myocardial infarction, and stroke.

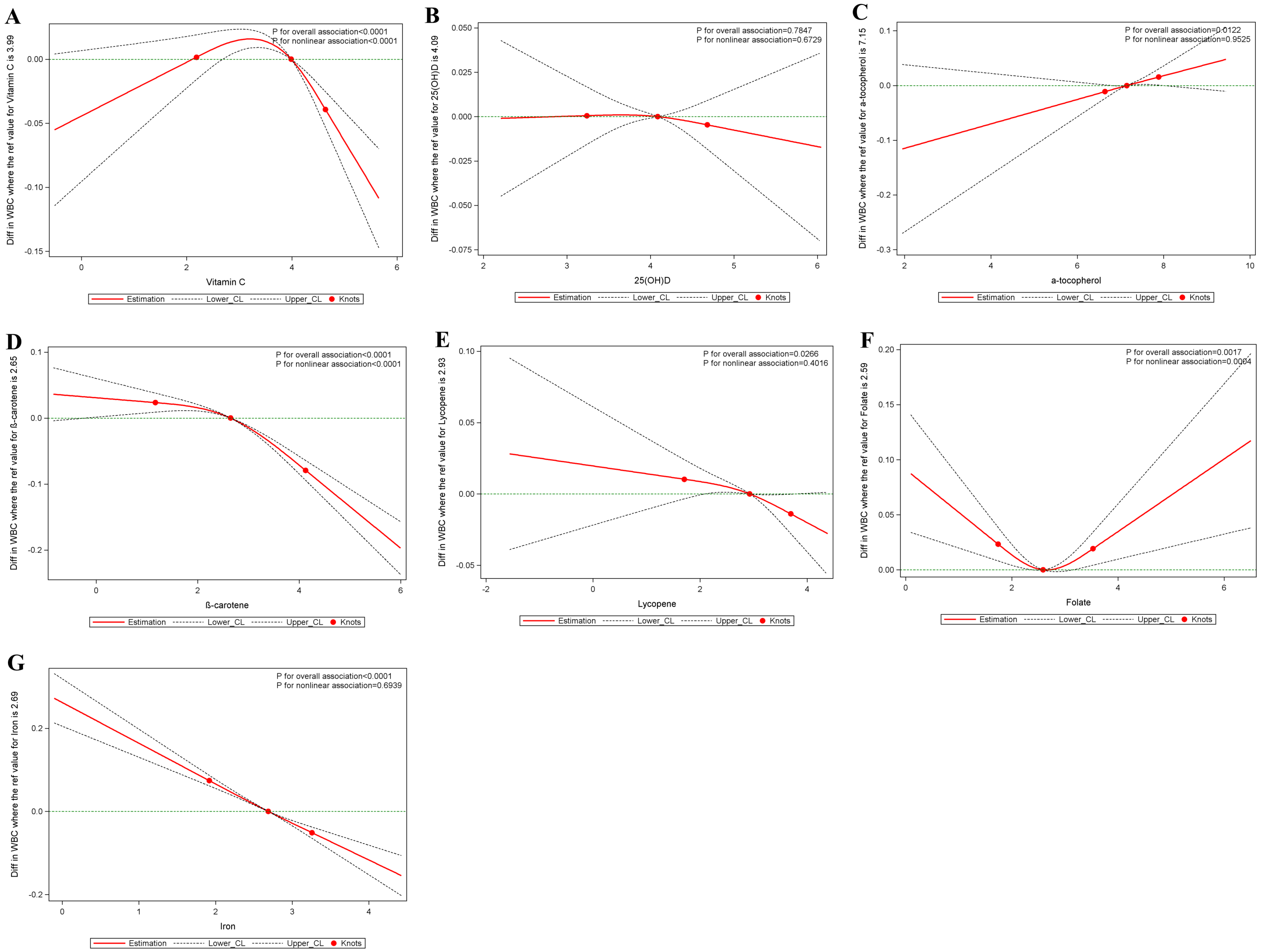

Supplementary Figure 3. Nonlinear dose-response associations between serum micronutrients and white blood cell count (WBC). All variables were log-transformed. Serum micronutrients were modeled using restricted cubic spline function with three knots located at the 5th, 50th, and 95th percentiles. Y represents the difference (Diff) in WBC for serum micronutrients, relative to their median values, which serve as the reference. Knots are indicated by dots. The model was adjusted for age, sex, race, body mass index, education, cigarette smoking, alcohol intake, cancer, hypertension, diabetes, heart failure, coronary heart disease, angina pectoris, myocardial infarction, and stroke.

Supplementary Table 1. Pearson correlations between serum micronutrients and inflammatory biomarkers among participants in the National Health and Nutrition Examination Survey in 2001-2006 and 2017-2018*

Serum micronutrients	Serum C-reactive protein			White blood cell count		
	n	r	p	n	r	p
Vitamin C	5,186	**-0.19**	**<0.0001**	8,356	**-0.13**	**<0.0001**
25(OH)D	7,891	**-0.16**	**<0.0001**	11,092	**-0.048**	**0.0009**
α-tocopherol	7,872	0.0077	0.59	11,002	-0.0001	0.99
β-carotene	7,868	**-0.28**	**<0.0001**	10,947	**-0.21**	**<0.0001**
Lycopene	7,865	**-0.12**	**<0.0001**	10,904	**-0.046**	**0.001**
Folate	7,903	**-0.071**	**<0.0001**	9,687	**-0.058**	**<0.0001**
Iron	7,875	**-0.28**	**<0.0001**	11,022	**-0.15**	**<0.0001**

* All variables were log-transformed. The Pearson correlation coefficient (r) was calculated by the %SURVEYCORRCOV Macro without adjustment of covariates.

Supplementary Table 2. Mediation analysis of white blood cell count for the associations between serum micronutrients and all-cause mortality, cancer mortality, and cardiovascular mortality among participants in the National Health and Nutrition Examination Survey in 2001-2006 and 2017-2018*

	Total effect		Indirect effect		Direct effect		Proportion
Serum micronutrients	Coefficients (95%CI)	p-value	Coefficients (95%CI)	p-value	Coefficients (95%CI)	p-value	Mediated# (%)
All-cause mortality							
Vitamin C	61.58 (52.21, 70.39)	<0.0001	1.25 (0.36, 2.34)	0.002	60.33 (51.20, 68.80)	<0.0001	**2.0**
25(OH)D	64.94 (60.56, 69.59)	<0.0001	0.34 (-0.31, 1.08)	0.31	64.61 (60.14, 69.31)	<0.0001	0.5
α-tocopherol	28.80 (18.02, 34.37)	<0.0001	-1.41 (-2.96, -0.29)	0.01	30.20 (20.16, 35.57)	<0.0001	NA
β-carotene	57.67 (45.56, 67.94)	<0.0001	3.04 (1.65, 4.54)	<0.0001	54.63 (42.55, 65.27)	<0.0001	**5.2**
Lycopene	58.53 (46.93, 68.91)	<0.0001	1.21 (0.36, 2.26)	<0.0001	57.32 (45.79, 67.56)	<0.0001	**2.0**
Folate	35.11 (11.16, 55.88)	0.010	0.19 (-1.19, 1.58)	0.76	34.91 (11.23, 55.80)	0.010	0.6
Iron	59.21 (37.46, 76.93)	<0.0001	8.65 (4.54, 13.08)	<0.0001	50.56 (26.46, 69.88)	0.002	**14.3**
Cancer mortality							
Vitamin C	257.73 (190.60, 333.81)	<0.0001	8.98 (3.60, 16.29)	<0.0001	248.75 (185.01, 321.81)	<0.0001	**3.4**
25(OH)D	181.73 (127.70, 247.83)	<0.0001	1.21 (-0.58, 3.71)	0.20	180.52 (126.64, 247.31)	<0.0001	0.6
α-tocopherol	115.61 (53.40, 153.45)	0.016	-2.26 (-12.12, 3.80)	0.50	117.87 (55.09, 155.97)	0.014	NA
β-carotene	303.74 (198.12, 420.60)	<0.0001	21.46 (8.00, 37.24)	<0.0001	282.28 (172.30, 400.26)	<0.0001	**7.0**
Lycopene	306.88 (213.71, 396.57)	<0.0001	10.21 (3.39, 19.61)	0.002	296.67 (205.09, 388.24)	<0.0001	**3.2**
Folate	366.57 (193.09, 532.34)	0.002	9.70 (0.88, 22.33)	0.026	356.87 (181.34, 517.42)	0.002	**2.4**
Iron	118.18 (-222.68, 347.14)	0.35	79.57 (33.65, 138.61)	<0.0001	38.61 (-332.84, 281.19)	0.66	35.6
Cardiovascular mortality							
Vitamin C	191.25 (128.28, 253.39)	<0.0001	5.74 (1.33, 12.17)	0.008	185.51 (125.07, 248.56)	<0.0001	**2.9**
25(OH)D	192.05 (156.84, 234.39)	<0.0001	0.56 (-1.27, 2.36)	0.47	191.49 (155.92, 233.61)	<0.0001	0.3
α-tocopherol	72.14 (-43.98, 118.24)	0.108	-6.87 (-17.85, -0.92)	0.006	79.01 (-35.68, 123.64)	0.090	NA
β-carotene	213.09 (140.72, 291.93)	<0.0001	12.12 (2.83, 23.84)	0.01	200.97 (127.69, 280.03)	<0.0001	**5.6**
Lycopene	217.68 (155.62, 280.54)	<0.0001	5.14 (0.95, 11.25)	0.012	212.54 (149.15, 274.34)	<0.0001	**2.3**
Folate	181.55 (37.97, 303.19)	0.006	4.10 (-0.91, 11.81)	0.13	177.46 (35.22, 297.93)	0.010	1.9
Iron	203.93 (82.20, 306.49)	0.006	26.69 (5.69, 51.55)	0.016	177.24 (44.34, 281.37)	0.010	**12.4**

CI: confidence interval.

* All serum micronutrients and white blood cell count were log-transformed. Causal mediation analysis was performed by adjusting for age, sex, race, body mass index, education, cigarette smoking, alcohol intake, cancer, hypertension, diabetes, heart failure, coronary heart disease, angina pectoris, myocardial infarction, and stroke. The coefficients represent the estimated change in the logarithm of survival time for each unit increase in serum micronutrients.

# NA: the proportion mediated was not computed when the point estimate of the direct effect was in the opposite direction to that of the indirect effect.
